# Prevalence and associated factors of delay antenatal care at public health institutions in Gondar city, Northwest Ethiopia, 2021: a cross-sectional study

**DOI:** 10.1186/s40834-022-00197-6

**Published:** 2023-01-16

**Authors:** Eshetu Abera, Jember Azanaw, Tsion Tadesse, Mastewal Endalew

**Affiliations:** 1 Department of Environmental and Occupational Health and Safety, Institute of Public Health, College of Medicine and Health Sciences, University of Gondar, Gondar, Ethiopia; 2 Departments of Clinical Midwifery, School of Midwifery, College of Medicine and Health Sciences, University of Gondar, Gondar, Ethiopia

**Keywords:** Antenatal care services, Pregnant mothers, Delayed antenatal care, Significant factors, Gondar

## Abstract

**Background:**

Antenatal care is critical for women’s and unborn children’s health. In Ethiopia there is still a delay in getting antenatal care visit in the first trimester as recommended by the World Health Organization. Therefore, the purpose of this study was to assess the prevalence of delayed antenatal care visits and associated factors among pregnant women who attend antenatal care at a public health facility in Gondar town, Northwest Ethiopia.

**Methods:**

An institutional-based cross-sectional study was conducted between August 20 to September 15/2021. A simple random sampling technique was used to select 392 women. Data were collected using a pre-tested structured questionnaire through a face-to-face interview. Epi Info version 7 and SSPS 26.0 were used for data entry and further analysis. Descriptive statistics and multivariable logistic regression analyses were performed. An adjusted odds ratio with 95% confidence interval at *p*-value < 0.05 was declared that the outcome can be statistically significant.

**Results:**

A total of 392 study participants with a response rate of 98% were participated. The mean age of study participants was 29.1 ± 6.5 (SD) years. In this study, the prevalence of delay antenatal care was 63.8%( 95% CI: 58.9, 68.9). Age (Adjusted odds ratio = 0.51; 95% CI: 0.28, 0.93), types of health facilities (Adjusted odds ratio = 2.02; 95% CI :( 1.12, 3.64**)**, and satisfaction with health service (Adjusted odds ratio = 3.23, 95%CI: (2.02, 5.16) were significantly associated with delay antenatal care.

**Conclusion:**

The current study found high prevalence of delay antenatal care. Age between 31 and 34, hospital health facility and satisfaction with health service quality were associated factors for delay antenatal care visit. To begin antenatal care follow-up in the recommended time frame, a collaborative effort between the Minister of Health, health facilities, and relevant stakeholders is needed.

## Introduction

Antenatal care is a type of prenatal care that is delivered to pregnant women with the primary goals of health promotion and sickness prevention, early detection and treatment of issues and existing conditions, and birth preparation and complication readiness planning [1].Women who attend their ANC appointments regularly are likely to have healthier pregnancies and less stressful births, whereas women who miss out this opportunity are regarded to be at risk of having difficult pregnancies [2]. The 2016 world health organization(WHO) antenatal care model recommends pregnant women should have a minimum of eight ANC contacts, with the first contact scheduled to take place in the first trimester (up to 12 weeks of gestation) [3].

Every day, about 1500 women die as a result of complications during pregnancy or childbirth around the world, with developing countries contributing to 98% of these deaths, with Sub-Saharan Africa accounting for half of all maternal deaths globally [4–6]. According to data from the 2016 Ethiopian Demographic Health Survey (EDHS), the maternal mortality rate in Ethiopia was 412 per 100,000 live births [7]. Based on WHO guidelines, four goal-oriented antenatal care(ANC) visits are an adequate and effective number of visits for providing essential interventions to pregnant women with no underlying health conditions, with the first visit occurring in the first trimester and resource-limited settings increasing the number of antenatal care visits to more than four has not been shown to enhance health outcomes in uncomplicated pregnancies [8], but the attendance of less than four ANC visits has been associated with an increased risk of prenatal mortality, particularly stillbirth [9].

Delays in antenatal care visits are still a problem, and study in Bangladesh, South Asia only 14% of pregnant women received their initial antenatal care within the first trimester [10]. Prevalence of delayed antenatal care has been documented in various African countries such as in the different districts of Zambia,72.0% and 86.6% [11, 12], Tanzania(70.4%) [13], Burkina Faso 62.93% [14], and Nigeria 65% [15]. According to, the Ethiopian Demographic Health Survey (EDHS) 2019 data, 43% of Ethiopian women had at least four antenatal care visits during their last pregnancy, whereas 26% had none. Only 28% of women had their first ANC visit during the first trimester, while 32% had their first visit during the fourth or fifth month of pregnancy, and 12% had their first visit during the sixth month of pregnancy. In the first trimester of pregnancy,43% of urban women received ANC, compared to 22% of rural women [16].

According to the findings of various studies, many factors influence the timing of antenatal care visits, including the maternal and husband’s education [14, 17], the types of occupation [18], the availability of health services [19], the number of children, and unwanted pregnancy [19], the household income level [14, 20], women’s employment [13], exposure to media[11, 21], age [12, 14, 21], marital status [13], and parity [12, 14], the previous experience of health service utilization [2], perceive the quality of service, and type pregnancy(planned or unplanned) [17, 22], were predictors of early antenatal care initiation. According to EDHS data from 2019, just 28% of women had their first ANC visit during the first trimester [16]. This indicated that there is still a gap in timely prenatal care bookings in the first trimester. Therefore, this study aimed to determine the prevalence of delayed antenatal care visits and associated factors among pregnant women who visited ANC at the public health facility in Gondar town, Northwest Ethiopia.

## Method

### Study design, area, and period

An institutional-based cross-sectional study was conducted between August 20 to September 15, 2021, in Gondar town public health institutions of Amhara regional state. The town has six sub-cities and 22 Keble (the lowest administrative level). The city has eight public health centers and one comprehensive specialized hospital.

### Study and source population

The source populations were all pregnant women who attended in selected public health centers, and hospitals and the study population were those who were selected for the study during the data collection period from those selected health institutions.

### Inclusion and exclusion criteria

All pregnant women attending ANC services in Gondar town public health institution were included in the study but pregnant women who were severely ill and unable to respond during data collection were excluding from the study.

### Sample size determination

The sample size was calculated using a single population proportion formula by using delay ANC from a similar study in Ethiopia, 62% [1], 95%CI (z α/2 = 1.96), a margin of error of 5%, and 10% non-response rate.

$$\text{n}=\frac{{(\text{Za}_{/2})}^{2}\text{*P}\left(1-\text{P}\right)}{\text{d}^2}\text{n}=\frac{\left(1.96\right)^2\text{*}0.62\left(0.38\right)}{{0.05}^2}=363,\;\mathrm{with}\;10\%\;\mathrm{non}-\mathrm{response}\;\mathrm{rate},\;\mathrm{final}\;\mathrm{sample}\;\mathrm{size}\;=\;400.$$

### Sampling procedure

**Fig. 1:**
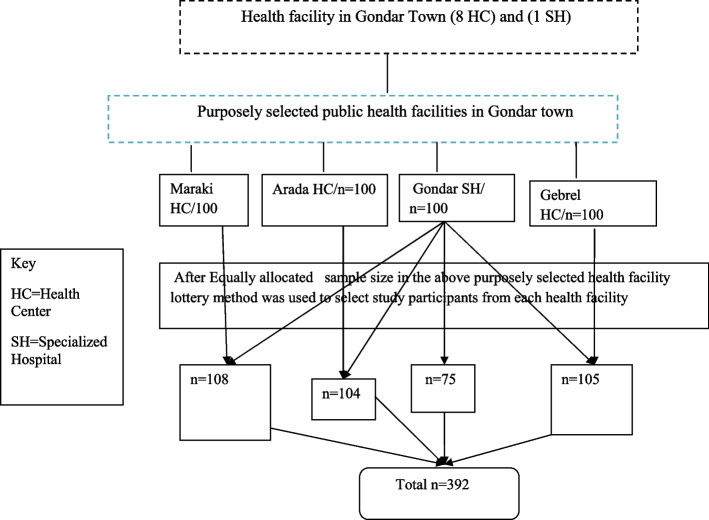
Equal allocation of sample size for each purposely selected health facility

In this study, purposive sampling procedure was used to selected health facilities, and simple random sampling technique such as lottery method were used to select the study participants within each health facilities(Fig. 1).

### Data collection tool and quality control

A structured interviewer–led questionnaire was prepared from reviewed articles [3, 23–25].The questionnaire has four components: The first components was Socio-demographic characteristics of respondents like age, level of education, residence, marital status, age at marriage, occupation, family size, and income. The second components were pregnant women’s history of ANC follow-ups like gravidity, gestational age during the first visit, history of abortion, and the number of ANC visits, and the third component was health service satisfaction-related questions and measured by using 13 questions with a series of simple statements with five-point Likert scales never to always. A pre-test was done and essential adjustments were performed.

The validity of the study tools was checked by the experts. Based on expertise’s suggestions, minor modifications were done and the final form was used for data collection. Krobach alpha value was computed and found 0.76. Trained MSC and MPH data collectors and supervisors were engaged in the data collection process. To assure the quality of the data, both the data collectors and the supervisors received two-day training on the study’s goal, data collection tool, data collection methods, checking the completeness of data collection tools, and maintaining confidentiality. Epi-Info version 7 software was used to code, enter, and clean the data, and transported to SPSS- 26 software for further analysis.

#### Operational definition

##### Delayed antenatal care

In this study, the outcome variable was defined based on world health organization recommendations at the time of the antenatal care visit made and delay ANC visit after the first trimester(after 12 weeks gestation) [3].

##### Public health institutions

In this study public health institution means those health institutions that governed by government and give service to the public and is not include private health institutions.

##### Types of health facility

In this study, the level of health facilities comprises hospitals and health centers.

##### Level of satisfaction

Those who answer the mean and above the mean value of the satisfaction survey questions were satisfied and those below the mean value were unsatisfied.

### Data management and analysis

The data was entered into EPI info version7 software and then exported to SPSS version 26 software for further analysis. Bivariable and multivariable logistic regression models were used to identify the association between delayed antenatal care visits and independent variables. The model fitness was assessed through Hosmer–Lemeshow goodness-of-fit test. *P*-value less than 0.05 and an odds ratio with 95% CI were used to declare the presence and the strength of association respectively.

## Results

### Socio-demographic characteristics of study participants

A total of 392 respondents with a response rate of 98.0% were participated in the study. The mean (± SD) age of respondents was 29.1(± 6.5) years with the highest (40.8%) proportion found in the range of 23–30 years. Nearly half (46.7%) of participants were unable to read and write. Most (85.2%) of the study participants were married. The majority of them were housewives (83.7%). The highest percentage (43.9%)) of the participants had monthly income in the range of 3000–5000 ETB (Table 1).

**Table 1:** Socio-demographic characteristics of study participants at Public Health facility in Gondar town, Northwest Ethiopia, 2021 (*n* = 392)

Variable		Frequency	Percent
Age	< 23	132	33.7
	23–30	160	40.8
	31- 34	85	21.7
	> 34	15	3.8
Marital status	Married	335	85.5
	Unmarried	57	14.5
Residence	Urban	348	88.8
	Rural	44	11.2
Mothers occupation	Housewife	328	83.7
	Merchant	30	7.7
	Government employee	30	7.7
	Student	4	1.0
Mothers educational status	Unable to read and write	183	46.7
	Able to read and write	27	6.9
	Primary school	117	29.8
	Secondary school	47	12.0
	Diploma and above	18	4.6
Household head	Father	249	63.5
	Mother	143	36.5
Level of income	< 3000	102	26.0
	3000–5000	172	43.9
	5001–6000	51	13.0
	> 6000	67	17.1

### Prevalence of delay antenatal care among pregnant women in Gondar town public health facility

From a total of 392 pregnant mothers who followed ANC, only 36.2% of respondents attended their ANC at the recommended time (within the first trimester), while 63.8% were delay from the first ANC visit (after the first trimester). The mean gestational age of booked respondents was 3.7 months, with a standard deviation of 1.2 months (Table 2).

**Table 2:** Prevalence of delay ANC among pregnant women in Gondar town public health facility (*n* = 392), 2021

ANC visit	Frequency	Percent	95% CI
After first trimester	250	63.8	( 58.9, 68.9)
Within first trimester	142	36.2	(31.1,41.1)

### Factors Associated with Delay antenatal care among women who visits ANC at public health institution in Gondar town

In bivariable logistic regression analysis, age of respondents, types of health facility, time taken to the health institution, and level of satisfaction with the health service were significant(*p*-value ≤ 0.2) factors for delay antenatal care visit, and exported to the multivariable binary logistic regression model. In multivariable binary logistic regression analysis, the age of respondents, types of health facility, and the level of satisfaction with health services quality were associated factors for pregnant women’s delay antenatal care visits. Participants between the ages of 31 and 34 were 49% less likely to delay from antenatal care visits when compared to participants who were less than 23 years (AOR = 0.51; 95% CI: 0.28, 0.93).

The odds of delay ANC visit were 2.02 times higher among pregnant women who were received antenatal care service in the hospital health institution, when compared to pregnant women who received antenatal care services in health center institutions (AOR = 2.02; 95% CI: (1.12,3.64)

The odds of delay ANC visit were 3.23 times higher among pregnant women who were satisfied with health services quality, when compared to pregnant women who were not satisfied with health service quality (AOR = 3.23, 95%CI: (2.02, 5.16) (Table 3).

**Table 3:** Factors Associated with delay antenatal care in Gondar town public health facilities (*n* = 392)2021

Variables		ANC follow up		COR,95%CI	AOR,95%CI	*p*- value
		Delay	Timely			
Age	< 23	95	37	1		
	23–30	104	56	0.72(0.44,1.19)	0.89(0.53,1.52)	0.68
	31–34	41	44	0.36(0.21,0.64)	**0.51(0.28,0.93)**	0.029
	> 34	10	5	0.78(0.25,2.43)	0.83(0.25,2.75)	0.76
Types of health facility	Health center	197	120	1	**1**	
	Hospital	53	22	1.46(0.85,2.53)	**2.02(1.12,3.64)**	0.019
Time taken	< 30 min	122	59	1	1	
	≥ 30 min	128	83	0.75(0.49,1.13)	0.74(0.47,1.16)	0.195
Level of satisfaction	Not satisfied	101	95	1	**1**	
	Satisfied	149	47	2.98(193,4.59)	3.23(2.02,5.16)*	0.001

Key: 1 = reference group,*p < 0.05; Hosmer– Lemeshow goodness-of-fit test value = 0.78

## Discussion

Based on this study finding, pregnant women who attend their antenatal care visit in Gondar town public health facilities were delay from timely antenatal care visit, and the study found that 63.8% of mothers did not begin their antenatal care follow-up during the first trimester as recommended by the world health organization. This finding is consistent with previous studies conducted in Southern Ethiopia [1] and northern Ethiopia(59%) [2] as well as 2016, Ethiopian, EDHS(67.61%) [26], and a systematic review and meta-analysis conducted in Ethiopia [27]. However, the finding of this study was lower than those found in Debre Berhan, Ethiopia (73.8%) [22], Nigeria (83%)[28], and Zambia, 86.6%[12]. and Tanzania(70.4%) [13]. On the other hand, this result is higher than that of research conducted in South Gondar, Ethiopia (52.5%) [29], Nigeria(2013, NDHS)(27%) [30]. This disparity may be attributed to differences in socio-demographic factors, study setting, and periods in which the study was conducted.

Regarding factors associated with delay antenatal care visits, the age of participants was found significantly associated with delay antenatal care visits. When compared to women under the age of 23 year, study participants found in the aged 31 to 34 were less likely to delay from their first antenatal care visit. This finding is in line with those from South Africa [31] and women less than 20 years of age were risk factors for delayed antenatal care, and a multi-country analysis in sub-Saharan African countries, and the odds of timely initiation of ANC was higher among women aged 25–34 years and greater than 35 years as compared to women aged 15–24 years [21]. This could be related to unique risk factors such as being pregnant for the first time and obtaining information or guidance on when to start antenatal care visits. In this study, other risk factors that had a significant association with delayed antenatal care visits was the types of health facilities, and the odds of delay ANC visit were higher among pregnant women who were received antenatal care service in the hospital health facility, when compared to pregnant women who received antenatal care services in health center facility. This might be due to health centers were easily accessible for pregnant women’s for their timely ANC follow up in their local village than hospital health facilities. This supported by study conducted in Rwanda and stated that delayed ANC would be lower among women seeking care from health posts because health posts are more geographically accessible by pregnant women than other types of health facilities [32].

The other important predictor variable that was found significantly associated with delayed antenatal care utilization was the level of satisfaction of women’s with health service quality delivered by the health institution, and in this study, pregnant women’s who were satisfied with healthcare services were more likely to delayed from timely ANC visit when compared to those who did not satisfied with healthcare service quality. This finding is supported by a study conducted in Jimma, stated that lower satisfaction level of pregnant women were attending focused antenatal care [16]. But this finding is inconsistent with study conducted in Northern Ethiopia and stated that participants who get adequate time for their previous antenatal care visit by health professionals that attending early compared to those who did not provide adequate time [33]. But the possible justification for this difference could be pregnant women’s who were satisfied with the health service quality of the health facility might be believe that they feel well being from the previous ANC visit satisfaction and this make them delay from timely initiation to start next pregnancy ANC to follow up.

## Conclusion

In conclusion prevalence of delayed antenatal care was high among pregnant women who attend antenatal care visits at public health facilities in Gondar town. Age of participants between 31 and 34 years, hospital health facility, and satisfaction with health services quality were associated factors for delayed antenatal care visits. To begin antenatal care visits within recommended time combined effort by the Minister of Health, health facilities, and concerned stakeholders are needed.

## Data Availability

The data used in this study is available at the corresponding author upon reasonable request.

## Abbreviations

AOR: Adjusted Odds Ratio
ANC: Antenatal Care
COR: Crude Odds Ratio
EDHS: Ethiopian Demography Health Survey
ETB: Ethiopian Birr
NDHS: Nigeria Demography Health Survey
SPSS: Statistical Package for Social Science
